# Factors influencing the deterioration from cognitive decline of normal aging to dementia among nursing home residents

**DOI:** 10.1186/s12877-020-01875-3

**Published:** 2020-11-18

**Authors:** Audai A. Hayajneh, Mohammad Rababa, Alia A. Alghwiri, Dina Masha’al

**Affiliations:** 1 Adult health-nursing department, Faculty of Nursing, Jordan University of Science and Technology, P.O. Box: 3030, Irbid, 22110 Jordan; 2 Department of Physiotherapy, School of Rehabilitation Sciences, University of Jordan, Amman, Jordan

**Keywords:** Cognitive decline, Normal aging, Dementia, Nursing homes residents, Impairment

## Abstract

**Background:**

A dearth of differential research exists regarding the determinants of mild cognitive impairment (MCI) and moderate cognitive impairment or dementia among nursing home residents. This study aimed to identify and examine the association between medical factors (number of comorbidities, hospitalization, disability, depression, frailty and quality of life) and moderate cognitive impairment or dementia in nursing homes residents.

**Methods:**

A cross-sectional design was used in this study. Convenience sampling of 182 participants was conducted in nursing homes located in the central part of Jordan. Montreal cognitive assessment (MoCA) was used to screen both MCI and moderate cognitive impairment or dementia. Bivariate analysis, including t-test and ANOVA test, and logistic and linear regression models were used to examine and identify the medical factors associated with moderate cognitive impairment or dementia compared to mild cognitive impairment.

**Results:**

Most nursing home residents had MCI (87.4%) compared to a few with moderate cognitive impairment or dementia. Age (t = − 2.773), number of comorbidities (t = − 4.045), depression (t = − 4.809), frailty (t = − 4.038), and quality of life physical (t = 3.282) and mental component summaries (t = 2.469) were significantly different between the stages of cognitive impairment. Marital status (t = − 4.050, *p* <  0.001), higher-income (t = 3.755, p <  0.001), recent hospitalization (t = 2.622,*p* = 0.01), depression (t = − 2.737, *p* = 0.007), and frailty (t = 2.852, *p* = 0.005) were significantly associated with mental ability scores among nursing home residents.

**Conclusion:**

The coexistence of comorbidities and depression among nursing home residents with MCI necessitates prompt management by healthcare providers to combat depressive symptoms in order to delay the dementia trajectory among at-risk residents.

**Trail registration:**

ClinicalTrials.gov NCT04589637, October 15,2020, Retrospectively registered.

**Supplementary Information:**

The online version contains supplementary material available at 10.1186/s12877-020-01875-3.

## Background

The decline in normal cognitive ability may be acute, chronic, or a sign of confusion related to delirium, dementia, major depression, or psychosis [1]. Mild cognitive impairment (MCI) is considered a transitional phase between normal cognition and dementia, in which customized interventions can be directed at this phase, aiming to stop its deterioration to dementia among older adults [2]. MCI is considered the “symptomatic predementia stage” on the gamut of cognitive decline [3].

Nursing homes are one of the places where older people or geriatric patients are institutionalized. Some institutionalized residents suffer from cognitive decline or cognitive impairment as well as dementia [4]. In addition to cognitive impairment [5], nursing home residents are at higher risk for frailty and dependency with aging [6]. Earlier studies showed that the prevalence rate of cognitive impairment in similar contexts ranged from 67 to 73% [5, 7, 8].

Cognitive impairment in nursing home residents should be focused upon because of higher severity compared to that of community-dwelling older adults [9]. Most of the nursing home residents in Jordan are not admitted based on specific medical admission criteria [10], which indicates that information is still limited regarding their healthcare needs as well as factors influencing their cognitive ability. Numerous factors were found to be associated with cognitive impairment or the risk of incidental dementia, such as frailty [11], depression [12], and health-related quality of life (HRQoL) [13, 14]. However, the factors of transition from mild cognitive impairment to moderate cognitive impairment stage or dementia among older adults have not been extensively understood in the literature.

Complete understanding of the stages of mild and moderate cognitive impairment, as concepts having their own attributes and features, significantly contributes to preventing and determining the trajectory of dementia as well as preparing management approaches for the admission of older individuals into nursing homes [15]. In light of this context, the aims of this study were to: 1) identify and examine the association between some selected medical factors (number of comorbidities, hospitalization, disability, depression, frailty and quality of life) and cognitive impairment or dementia in nursing homes residents, and 2) examine the association between the life course determinants (age, sex, education, income, and marital status) and moderate cognitive impairment or dementia.

## Methods

### Study design and setting

A cross-sectional design was used in the current study. Convenience sampling of 182 participants was used to obtain moderate size correlation (r = 0.35), given a power of 0.80, two-sided Type I error rate of *p* = 0.0167, based on nQuery calculation [16]. The participants were recruited from 5 holistic nursing homes, including private and public nursing homes, located in the center of Jordan. Inclusion criteria were as follows: Jordanian participants, aged 60 years and older, and living in nursing homes. Exclusion criteria were neurological disorders, such as stroke or brain injury.

### Instruments

A demographic and health survey was developed by the principal investigator, including age, sex, education level, household income, marital status, impaired activities of daily living, the number of comorbidities, and the number of hospitalizations during the year before (See Additional file 1). The Arabic version-Montreal Cognitive Assessment (MoCA) tool was used to detect both mild and moderate cognitive impairment based on its total score [17] (See Additional file 1). It has 10 items about different cognitive domains (executive functions, memory, language, attention and concentration, conceptual thinking, calculations, visuoconstructional skills, and orientation). The total score of the test is a 30-point score and it takes 10 min to administer. A score of 26 or above indicates no cognitive impairment. The sensitivity of the cutoff score to detect MCI is 90% and to detect mild Alzheimer’s disease is 100%, and its specificity has been reported to be excellent (87%) [18]. A total score of 18 to 25 was considered mild cognitive impairment, and a score of 10 to 17 was considered moderate cognitive impairment [17] or dementia. The MoCA tool was developed by Dr. Nasreddine and colleagues (2005), who has extensively used this tool in detecting cognitive impairment in nursing home residents. Using the MoCA has been well-documented in the literature to determine mild and moderate cognitive impairment [17]. In conclusion, the MoCA is the most precise cognitive ability measure that differentiate between the stages of cognitive impairment and had excellent sensitivity and specificity values to detect MCI and mild Alzheimer’s disease [18] compared to other cognitive ability measures.

The Arabic version-geriatric depression scale (GDS) was used to detect depression and it has 15 items. The total score was 15 and its scores were distributed as follows: 0–4 normal, 5–8 mild depression, 9–11 moderate depression, and 12–15 severe depression [19]. Greenberg’s study revealed that the GDS-15 had high sensitivity and specificity of 92 and 89%, respectively [19] (See Additional file 1).

The Arabic version-HRQoL (Short Form (SF-36)) was used in the current study. The SF-36 health survey entails 2 main domains (physical and mental). The SF-36 health survey has 36 questions, including 8 categories: physical functioning (PF), general health (GH), vitality (V), social functioning (SF), role-physical (RP), bodily pain (BP), mental health (MH), and role-emotional (RE). Each category has a 0–100 scale calculated using a special software. Lower score indicates more disability, and a higher score shows less disability/higher HRQoL in its category [20]. The scores of the Arabic-SF-36v2® were calculated using Qualtrics Software (Medical Outcomes Trust) (See Additional file 1).

The Arabic version-Tilburg frailty indicator (TFI) was used in the current study to assess frailty among Jordanian older adults. The original TFI created by Gobbens and colleagues [21] has 15 items, entailing 3 domains: physical, psychological, and social domains. The reliability (KR 20 = 0.77) and convergent and divergent validity of the Arabic version-TFI have been established in Jordanian population [22]. The TFI is the only frailty instrument that has been translated and validated to use in Jordanian older adult population [22]. It has only fifteen questions [21], which is easy to administer in older adult population (See Additional file 1).

### Data collection

After institutional review board (IRB) approval (#20180229) from both the Jordan University of Science and Technology and targeted nursing homes, participants of the study were recruited through nursing home visits and asked to participate in the study. An overseeing mental health expert have ruled that all participants have been deemed capable of ethically and medically consenting for their participation in the study. Consent forms were delivered to or read aloud to the older adults prior to collecting data from them. To avoid drift resulting from different data collectors, a training session on how to gain information using the instruments used in the current study was conducted for data collectors.

### Statistical analysis

Bivariate analysis, including t-test and ANOVA test, was used to examine the correlations between health variables and mental ability. Thereafter, multivariate analysis, including logistic and linear regression models was conducted, for the correlations between health variables and both mild and moderate cognitive impairment. The statistical analysis was performed using the Statistical Package for Social Sciences (SPSS) version 25 (SPSS, Inc., Chicago, Ill).

## Results

Altogether, 182 nursing home residents participated in the current study. Most of them had MCI (87.4%) compared to a few with moderate cognitive impairment or dementia. Their mean age was 64.42 years (SD = 10.32) and most of them were male (91.3%). Only 24 participants were not married (13%). The largest percentage of the participants included residents with secondary school education (18.7%). Most of the participants who answered the question about monthly income had less than 450 JOD (USD 630). The number of governmental nursing homes residents was 103 (56.3%). Only 3.3% of the participants had disability and 73.8% had frailty.

The mean depression total score was 6.148 (SD = 3.644). One hundred and sixty participants had mild cognitive impairment (87.4%) and the rest had moderate cognitive impairment (12.6%). Most of the 8 components of the SF36 health survey were approximately 50% on the scale, which represents a range from 0 (worst quality of life) to 100% (highest quality of life). Two main domains of HRQoL (physical and mental) among participants were 47 and 46%, respectively (Table 1).

**Table 1:** Social-Demographic and health characteristics of nursing homes residents (*N* = 182)

	N (%)	Mean (SD)
**Age**		64.42 (10.32)
**Sex**		
Male	167 (91.3)	
Female	15 (8.7)	
**Marital Status**		
Married	158 (86.8)	
Non married	24 (13.2)	
**Education**		
Illiterate	22 (12.1)	
Non-Illiterate	160 (87.9)	
**Income (Jordanian Dinars)**		
< 450	172 (94.5)	
> 450	10 (5.5)	
**Type of nursing homes**		
Governmental	103 (56.6)	
Private	79 (43.4)	
**The number of comorbidities**		1.93 (1.59)
**Disability**		
Yes	6 (3.3)	
No	176 (96.7)	
**Hospitalization**		
Yes	45 (29.5)	
No	128 (70.5)	
**Depression (GDS)**		6.148 (3.644)
**Frailty (TFI)**		7.17 (2.48)
Frail	134 (73.8)	
Non frail	48 (26.2)	
**Cognitive Impairment (**MoCA)		20.17(2.3)
Mild	159 (87.4)	
Moderate (dementia)	23 (12.6)	
**Quality of Life (SF36)**		
Physical Functioning (PF)		37.77 (8.82)
Role Physical (RP)		47.72 (8.23)
Bodily Pain (BP)		52.77 (8.13)
General Health (GH)		49.31 (9.25)
Vitality (VT)		46.95 (8.84)
Social Functioning (SF)		48.08 (7.95)
Role Emotional (RE)		45.50 (9.53)
Mental Health (MH)		42.58 (7.10)
**Physical Components Summary**		**47.00 (7.73)**
**Mental Components Summary**		**46.03 (7.09)**

Note: GDS: Geriatric depression scale, TFI: Tilburg frailty indicator, MoCA: Montreal cognitive assessment

Table 2 displays the associations between the demographic and health characteristics of nursing home residents and the stages of mild and moderate cognitive impairment. Residents were significantly older in the moderate (mean = 68.8, SD =13.03) than in the mild (mean = 63.8, SD = 9.79) cognitive impairment group (t = − 2.773, *p* = 0.031). The number of comorbidities was higher in the moderate group (mean = 3.18, SD = 2.22) than in the mild cognitive impairment group (t = − 4.045, *p* <  0.001). Nursing home residents who were hospitalized during the year before were likely to have moderate cognitive impairment (*p* = 0.016) than those who were not hospitalized (23.07 to 7.75%).

**Table 2:** Bivariate analysis for the associations between demographic and health characteristics of nursing homes residents and the stages of mild and moderate cognitive impairment (*N* = 182)

	Statistic tests	Mean (SD) or n (%) Cognitive Impairment Mild (160) vs. Moderate (22)		df	***p***-value
**Age**	t = −2.773	63.8 (9.79)	68.8 (13.03)		**0.031**
**Sex**					
Female	Χ^2^ = 0.024	13 (86.67)	2 (13.33)	1	0.877
Male		147 (88.02)	20 (11.98)		
**Marital Status**					
Married	Χ^2^ = 36.7	22 (91.68)	2 (8.32)	1	0.545
Non-married		138 (87.34)	20 (12.66)		
**Education**					
Illiterate Non illiterate	Fisher’s = 0.875	18 (81.82) 142 (88.75)	4 (81.18) 18 (11.25)		0.303
**Income (Jordanian dinars)**					
< 450JOD	Fisher’s = 0.04	151 (87.80)	21 (12.20)		0.835
> 450JOD		9 (90)	1 (10)		
**Type of nursing homes**					
Governmental	Χ^2^ = 0.505	71 (89.87)	8 (10.13)		0.47
Private		89 (86.40)	14 (13.60)		
**The number of comorbidities**	t = − 4.045	1.75 (1.414)	3.18 (2.22)		**< 0.001**
**Hospitalization**					
Hospitalized during last year	Χ^2^ = 8.329	40 (76.92)	12 (23.08)	2	**0.016**
Non-hospitalized during last year		119 (92.25)	10 (7.75)		
**Disability**					
With disability Without disability	Χ^2^ = 0.303	4 (80) 156 (88)	1 (20) 21 (12)	1	0.582
**Depression**	t = − 4.809	5.69 (3.36)	9.45 (3.95)	180	**< 0.001**
**Frailty**	t = − 4.038	7.46 (3.79)	10.86 (2.95)		**< 0.001**
**Quality of life**					
Physical Functioning	t = 3.605	38.62 (8.51)	31.61 (8.79)	180	**< 0.001**
Role Physical	t = 1.659	48.10 (7.89)	45.01 (10.2)	180	0.09
Bodily Pain	t = 2.645	53.35 (7.43)	48.54 (11.3)	180	**0.009**
General Health	t = 3.451	50.17 (8.64)	43.11 (11.28)	180	**< 0.001**
Vitality	t = 2.656	47.59 (8.54)	42.33 (9.82)	180	**0.009**
Social Functioning	t = 3.291	48.78 (7.15)	42.98 (11.3)	180	**0.001**
Role Emotional	t = 2.062	46.04 (9.01)	41.6 (12.28)	180	**0.041**
Mental Health	t = 2.318	43.03 (6.98)	39.33 (7.25)	180	**0.022**
Physical Components Summary	t = 3.282	47.68 (7.46)	42.07 (8.03)	180	**0.001**
Mental Components Summary	t = 2.469	46.50 (6.52)	42.57 (9.82)	180	**0.041**

Depression in nursing home residents was significantly higher in the moderate (mean = 9.45, SD = 3.95) than in the mild (mean = 5.69, SD = 3.36) cognitive impairment group (t = − 4.809, *p* <  0.001). Pertaining to frailty, nursing home residents had significantly more frailty in the moderate (mean = 10.86, SD = 2.95) than in the mild (mean = 7.46, SD = 3.79) cognitive impairment group (t = − 4.038, p <  0.001). The two main branches of HRQoL (physical and mental) as well as their components, were significantly better in the mild compared to the moderate cognitive impairment group (*p* <  0.05), except for the role-physical component, which was not significant (*p* = 0.09) (Table 2).

A logistic regression model using a stepwise regression method was used to identify and explore coexisted factors of moderate cognitive impairment among nursing home residents. Only significant variables after bivariate analysis were chosen to build the primary model for the coexisting factors of the moderate cognitive impairment stage. These variables were age, the number of comorbidities, hospitalization, depression, frailty, and physical and mental component summaries for quality of life (Table 3). The indices of goodness of fit for the yielded logistic regression model are shown in Table 4, which were considered acceptable.

**Table 3:** Logistic regression model of predictors of moderate vs. mild cognitive impairment

Model	Parameter	Estimate	Standard Error	Adjusted Odds Ratio	p	Confidence Interval Lower Upper bound bound	
1	(Intercept)	−1.984	0.227	0.138	< .001	−2.430	−1.538
2	(Intercept)	−4.043	0.619	0.018	< .001	−5.257	−2.829
	Depression	0.275	0.066	1.317	< .001	0.146	0.404
3	(Intercept)	−4.319	0.667	0.013	< .001	−5.626	−3.011
	Depression	0.223	0.073	1.250	0.002	0.081	0.365
	The number of comorbidities	0.271	0.149	1.312	0.068	−0.020	0.563
4	(Intercept)	−1.554	1.955	0.211	0.427	−5.387	2.278
	Depression	0.265	0.080	1.304	**< .001**	0.109	0.422
	The number of comorbidities	0.432	0.187	1.540	**0.021**	0.065	0.798
	Age	− 0.052	0.036	0.949	0.146	−0.123	0.018

Note: Stepwise regression method

**Table 4:** The indices of goodness of fit for the yielded logistic regression model

Model	AIC	BIC	df	ΔΧ^**2**^	p
1	136.197	139.401	181		
2	118.525	124.933	180	19.672	< .001
3	117.347	126.959	179	3.178	0.075
4	117.183	129.999	178	2.165	0.141

Model 4 (Table 3) yielded two significant medical factors of moderate cognitive impairment, depression (*p* <  0.001) and the number of comorbidities (*p* = 0.021). The indices of model 4 showed adequate fit based on the non-significant *p* value of the chi-square test (*p* = 0.141) and low values of both AIC and BIC. AIC and BIC values represented the lowest error measurements values of 117.183 and 129.999, respectively. Based on model 4, for any additional score increases on the depression scale, the odds of moderate cognitive impairment increased by 30.4%. Moreover, for any additional comorbidity among nursing home residents, the odds of moderate cognitive impairment increased by 54%. However, age was not significant in Model 4 (Table 3).

Linear regression models of life course determinants and medical factors have been shown in Tables 5 and 6, respectively. Among nursing home residents, marital status (t = − 4.050, *p* <  0.001), higher-income (t = 3.755, *p* < 0.001), recent hospitalization (t = 2.622,*p* = 0.01), depression (t = − 2.737, *p* = 0.007), and frailty (t = 2.852, *p* = 0.005) were significantly associated with low mental ability.

**Table 5:** Linear regression model one of covariate variables of mental ability

Model		Standardized	t	p
1	(Intercept)		19.685	< .001
	Age	−0.198	−2.236	**0.027**
	Sex	−0.035	−0.547	0.585
	Marital status	−0.258	−3.990	**< .001**
	Education	−0.018	−0.275	0.783
	Income	0.185	2.902	**0.004**
	Type of nursing home	−0.088	−1.334	0.184
	The number of comorbidities	−0.274	−3.137	**0.002**

Model 1 R^2^ = 0.30, Adjusted R^2^ = 0.274, p = < 0.001

**Table 6:** Linear regression model two of covariate variables and predictors of mental ability

Model		Standardized	t	p
2	(Intercept)		7.113	< .001
	Age	0.057	0.625	0.533
	Sex	0.012	0.204	0.838
	Marital status	− 0.237	−4.050	**< .001**
	Education	−0.015	−0.251	0.802
	Income	0.221	3.755	**< .001**
	Type of nursing home	−0.012	−0.186	0.852
	The number of comorbidities	−0.048	−0.537	0.592
	Hospitalization	0.183	2.622	**0.010**
	Disability	0.019	0.313	0.755
	Depression	−0.240	−2.737	**0.007**
	Frailty	−0.283	−2.852	**0.005**
	PCS	−0.016	−0.210	0.834
	MCS	0.044	0.624	0.533

Model 2 R^2^ = 0.46, Adjusted R^2^ = 0.414, p = < 0.001

## Discussion

The main aim of this study was to identify the factors that lead to the conversion from MCI to moderate cognitive impairment or dementia in nursing home residents. The reason of very young nursing home residents in our study is that older adulthood in Jordan is most commonly defined at age of 55 years [23, 24]. Pertaining to inequal sexes in our sample, cultural considerations and social stigma play a genuine role in determining where older adults are living, and who care for them [10, 25, 26] or discouraging Arab families from admitting their older relatives to nursing homes [27]. This could explain the small percentage of female nursing home participants in our study as it might be unacceptable for Jordanian female older adults to be admitted to nursing homes. Based on the logistic regression model, depression and the number of comorbidities were found to be significantly associated with moderate cognitive impairment or dementia in this population. Depression contributes significantly to dementia through its effects on the dementia trajectory [28]. This contribution has been reported in a previous recent study [28]. This is in line with the findings of Makizako and colleagues [29]. Their study reported that the coexistence of depression and MCI significantly predisposes older adults to dementia.

Similar to our results in the current study, depression was found to be common among patients with MCI and cognitive deficits [30], placing those patients at higher risk for dementia in their later years. This combination of depression and MCI complicates the health condition of these patients at later stages of life, particularly affecting their treatment for combating cognitive decline, such as donepezil for Alzheimer’s disease as reported by Devanand and colleagues [31].

The negative effect on the treatment approaches managing cognitive decline is not the only health challenge among these patients. The presence of comorbidities, such as diabetes mellitus, in addition to depression and MCI among older adults, necessitates early assessment and proper management to achieve better quality of life [32]. In our study, HRQoL was significantly correlated with the stages of cognitive impairment. This confirms the findings of the study by Liu and colleagues [33], in which MCI alone or with other comorbidities was found to be significantly and negatively associated with HRQoL.

In the current study, the number of comorbidities was significantly associated with moderate cognitive impairment or dementia, which contributes to progression of MCI to dementia among nursing home residents. Numerous comorbidities in the literature have been found to be associated with MCI and dementia, such as cerebrovascular disease, cirrhosis, asthma, and diabetes mellitus [34]. Dementia severity is also dependent on the severity of each comorbidity [35]. However, this is not in accordance with a previous report [36] that revealed that the prevalence of comorbidities, except anemia, was similar across different cognitive impairment stages. Further research is needed to explore the extent to which each comorbidity contributes to cognitive impairment among nursing home residents.

According to linear regression models in the current study, marital status was significantly associated with mental ability. This is in concordance with another study [37] that reported older individuals living alone are at higher risk for common mental disorders, and this justifies that single or unmarried residents might have low mental ability. Therefore, it places them at higher risk for moderate cognitive impairment or dementia. Nursing home residents with higher income were found to have higher mental ability scores in our study. A similar report [38] suggested that poverty increases the risk of mental ability decline, and consequently, mental illness. This indicates how socio-economic status might contribute to changing mental ability scores towards moderate cognitive impairment or dementia. Recent hospitalizations were found to be associated with low mental ability in the current study. It is difficult to determine the relationship between length of hospital stay and cognitive decline, especially among older people because of multiple risk factors affecting their mental status during hospitalization, such as drug effects, surgery, stress, and discharge from hospital [39]. In addition, other diseases, such as anemia may influence the cognitive function in older adults during hospitalization [40]. Lastly, frailty was also significantly associated with a low mental ability score in our study. This is in concordance with a report [41] stating that frailty predisposes older individuals aged 68 years and above to incidental dementia. This outcome could help in developing interventions tailored to nursing home residents with frailty prior to the development of dementia.

Although age was found to be significantly and negatively correlated with stages of cognitive impairment in the bivariate analysis of the current study, it was not a predictor of moderate cognitive impairment or dementia. This corroborates with established literature that normal aging changes are totally different from moderate cognitive impairment or dementia. MCI represents the intermediate phase between normal aging and dementia, where novel early interventions can be rendered by healthcare providers [42].

The ability to differentiate between normal aging and neurodegenerative etiology of the brain can contribute significantly to implementing the required management approach in a timely manner and delay additional cognitive decline [43] or developing dementia. Recognizing dementia-specific factors assists healthcare providers, including nurses, in setting up highly flexible and sensitive interventions, treatments, and protocols to meet patients’ needs in nursing homes [44–46]. Further research should be conducted to address the extent to which we can consider cognitive changes that accompany normal aging and to what extent these changes are related to pathological conditions. Our study is not without limitations that are inherent in the cross-sectional design. However, the findings of this study shed light on the importance of comprehensive examination of nursing home residents, including detecting depression symptoms as well as documenting all existing comorbidities to design a specific plan of treatment that halts the deterioration of their cognitive abilities.

## Conclusion

Being single, low income, recent hospitalization, depression, and frailty were significantly correlated with low mental ability, moderate cognitive impairment, or dementia among nursing home residents, leading to the progression of cognitive impairment. The age of nursing home residents was not a significant associated with moderate cognitive impairment or dementia. The coexistence of such medical factors among nursing home residents with MCI necessitates prompt management by healthcare providers in order to delay the dementia trajectory among those at-risk.

## Supplementary Information

**Additional file 1.**

## Data Availability

The datasets used and/or analyzed during the current study available from the corresponding author on reasonable request.

## Abbreviations

MCI: Mild Cognitive Impairment
MoCA: Montreal Cognitive Assessment
GDS: Geriatric Depression Scale
