# Supplementary material for: Factors influencing the deterioration from cognitive decline of normal aging to dementia among nursing home residents
Source: BMC Geriatr. 2020 Nov 18;20:479. doi: 10.1186/s12877-020-01875-3 (PMC7672837; doi:10.1186/s12877-020-01875-3)

**APPENDIX A**

**THE ENGLISH VERSION OF DEMOGRAPHIC/HEALTH VARIABLES**

**Demographics:**

1 What is your age?

_ _ Code age in years

0 7 Don’t know / Not sure

0 9 Refused

2 Are you?

0 Male

1 Female

3 Are you…?

1 Married

2 Divorced

3 Widowed

4 Separated

5 Never married

4 How many **family members live in your household?**

5 What is the highest grade or year of school you completed?

1 Never attended school or only attended kindergarten

2 Grades 1 through 8 (Elementary)

3 Grades 9 through 11 (Some high school)

4 Grade 12 or GED (High school graduate)

5 College 1 year to 3 years (Some college or technical school)

6 College 4 years or more (College graduate)

9 Refused

7 Monthly household income:

1 Less than 250 JD

2 between 250-350 JD

3 between 350-450 JD

4 between 450-550 JD

5 between 550-650 JD

6 between 650-750 JD

7 between 750-850 JD

8 between 850-950 JD

9 over 950 JD

7 7 Don’t know / Not sure

9 9 Refused

**Demographics (cont.)**

7.8 Have you ever been admitted to hospital in the past year and how many times?

1 Yes and how many ……

2 No

8.1 Have you had any type of disabilities?

1 Yes and it is ……

2 No

**Health variables:**

| 4 Refused | 3 Don’t know / Not sure | **2 No** | **1 Yes** |  |
| --- | --- | --- | --- | --- |
|  |  |  |  | (Ever told) you had a **hypertension?** |
|  |  |  |  | (Ever told) you had an a**ngina, heart attack, or coronary heart disease?** |
|  |  |  |  | (Ever told) you had a **stroke**? |
|  |  |  |  | (Ever told) you had **asthma**? |
|  |  |  |  | (Ever told) you **have Chronic Obstructive Pulmonary Disease or COPD, emphysema or**  **chronic bronchitis**? |
|  |  |  |  | (Ever told) you have some form of **arthritis, rheumatoid arthritis, or gout**? |
|  |  |  |  | Do you have any **trouble seeing, even when wearing glasses or contact lenses**? |
|  |  |  |  | Do you have any **hearing problem?** |
|  |  |  |  | (Ever told) you have **diabetes**? |

**APPENDIX B**

**MONTREAL COGNITIVE ASSESSMENT TOOL/ENGLISH VERSION**


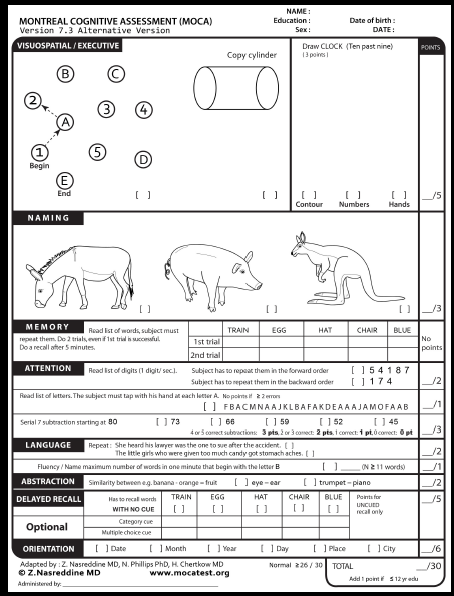


**APPENDIX C**

**THE GERIATRIC DEPRESSION SCALE: SHORT FORM**

Choose the best answer for how you have felt over the past week:

1. Are you basically satisfied with your life? YES / NO

2. Have you dropped many of your activities and interests? YES / NO

3. Do you feel that your life is empty? YES / NO

4. Do you often get bored? YES / NO

5. Are you in good spirits most of the time? YES / NO

6. Are you afraid that something bad is going to happen to you? YES / NO

7. Do you feel happy most of the time? YES / NO

8. Do you often feel helpless? YES / NO

9. Do you prefer to stay at home, rather than going out and doing new things? YES / NO

10. Do you feel you have more problems with memory than most? YES / NO

11. Do you think it is wonderful to be alive now? YES / NO

12. Do you feel pretty worthless the way you are now? YES / NO

13. Do you feel full of energy? YES / NO

14. Do you feel that your situation is hopeless? YES / NO

15. Do you think that most people are better off than you are? YES / NO

Answers in bold indicate depression. Score 1 point for each bolded answer.

A score > 5 points is suggestive of depression.

A score ≥ 10 points is almost always indicative of depression.

A score > 5 points should warrant a follow-up comprehensive assessment.

Source: http://www.stanford.edu/~yesavage/GDS.html

This scale is in the public domain.

**APPENDIX D**

**THE HEALTH RELATED QUALITY OF LIFE (SF-36)**

**_____________________________________________________________**

**Your Health and Well-Being**

**This survey asks for your views about your health. This information will help keep track of how you feel and how well you are able to do your usual activities. *Thank you for completing this survey!***

**For each of the following questions, please mark an  in the one box that best describes your answer.**

**1. In general, would you say your health is:**

| Excellent | Very good | Good | Fair | Poor |
| --- | --- | --- | --- | --- |
|  |  |  |  |  |
| 1 | 2 | 3 | 4 | 5 |

**2. Compared to one year ago, how would you rate your health in general now?**

| Much better now than one year ago | Somewhat better  now than one year ago | About the same as  one year ago | Somewhat worse  now than one year ago | Much worse now than one year ago |
| --- | --- | --- | --- | --- |
|  |  |  |  |  |
| 1 | 2 | 3 | 4 | 5 |

**3. The following questions are about activities you might do during a typical day. Does your health now limit you in these activities? If so, how much?**


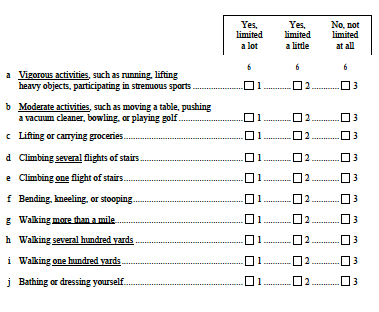


**4. During the past 4 weeks, how much of the time have you had any of the following problems with your work or other regular daily activities as a result of your physical health?**

|  | All of  the time | Most of  the time | Some of  the time | A little of the time | None of  the time |
| --- | --- | --- | --- | --- | --- |
|  |  |  |  |  |  |
| a Cut down on the amount of  time you spent on work or  other activities  1  2  3  4  5 | | | | | |
| b Accomplished less than you  would like  1  2  3  4  5 | | | | | |
| c Were limited in the kind of  work or other activities  1  2  3  4  5 | | | | | |
| d Had difficulty performing the  work or other activities (for  example, it took extra effort)  1  2  3  4  5 | | | | | |

**5. During the past 4 weeks, how much of the time have you had any of the following problems with your work or other regular daily activities as a result of any emotional** **problems (such as feeling depressed or anxious)?**

|  | All of  the time | Most of  the time | Some of  the time | A little of the time | None of  the time |
| --- | --- | --- | --- | --- | --- |
|  |  |  |  |  |  |
| a Cut down on the amount of  time you spent on work or  other activities  1  2  3  4  5 | | | | | |
| b Accomplished less than you  would like  1  2  3  4  5 | | | | | |
| c Did work or other activities  less carefully than usual  1  2  3  4  5 | | | | | |

**6. During the past 4 weeks, to what extent has your physical health or emotional problems interfered with your normal social activities with family, friends, neighbors, or groups?**

| Not at all | Slightly | Moderately | Quite a bit | Extremely |
| --- | --- | --- | --- | --- |
|  |  |  |  |  |
| 1 | 2 | 3 | 4 | 5 |

**7. How much bodily pain have you had during the past 4 weeks?**

| None | Very mild | Mild | Moderate | Severe | Very severe | |
| --- | --- | --- | --- | --- | --- | --- |
|  |  |  |  |  |  |  |
| 1 | 2 | 3 | 4 | 5 | 6 |  |

**8. During the past 4 weeks, how much did pain interfere with your normal work (including both work outside the home and housework)?**

| Not at all | A little bit | Moderately | Quite a bit | Extremely |
| --- | --- | --- | --- | --- |
|  |  |  |  |  |
| 1 | 2 | 3 | 4 | 5 |

**9. These questions are about how you feel and how things have been with you during the past 4 weeks. For each question, please give the one answer that comes closest to the way you have been feeling. How much of the time during the past 4 weeks…**

|  | All of  the time | Most of  the time | Some of  the time | A little of the time | None of  the time |
| --- | --- | --- | --- | --- | --- |
|  |  |  |  |  |  |
| a Did you feel full of life?  1  2  3  4  5 | | | | | |
| b Have you been very nervous?  1  2  3  4  5 | | | | | |
| c Have you felt so down in the  dumps that nothing could  cheer you up?  1  2  3  4  5 | | | | | |
| d Have you felt calm and  peaceful?  1  2  3  4  5 | | | | | |
| e Did you have a lot of energy?  1  2  3  4  5 | | | | | |
| f Have you felt downhearted  and depressed?  1  2  3  4  5 | | | | | |
| g Did you feel worn out?  1  2  3  4  5 | | | | | |
| h Have you been happy?  1  2  3  4  5 | | | | | |
| i Did you feel tired?  1  2  3  4  5 | | | | | |

**10. During the past 4 weeks, how much of the time has your physical health or emotional problems interfered with your social activities (like visiting with friends, relatives, etc.)?**

| All of  the time | Most of  the time | Some of  the time | A little of  the time | None of  the time |
| --- | --- | --- | --- | --- |
|  |  |  |  |  |
| 1 | 2 | 3 | 4 | 5 |

**11. How TRUE or FALSE is each of the following statements for you?**

|  | Definitely true | Mostly  true | Don’t  know | Mostly  false | Definitely false |
| --- | --- | --- | --- | --- | --- |
|  |  |  |  |  |  |
| a I seem to get sick a little easier than other people  1  2  3  4  5 | | | | | |
| b I am as healthy as  anybody I know  1  2  3  4  5 | | | | | |
| c I expect my health to  get worse  1  2  3  4  5 | | | | | |
| d My health is excellent  1  2  3  4  5 | | | | | |

***Thank you for completing these questions!***

**APPENDIX I**

**THE TILBURG FRAILTY INDICATOR (TFI)**


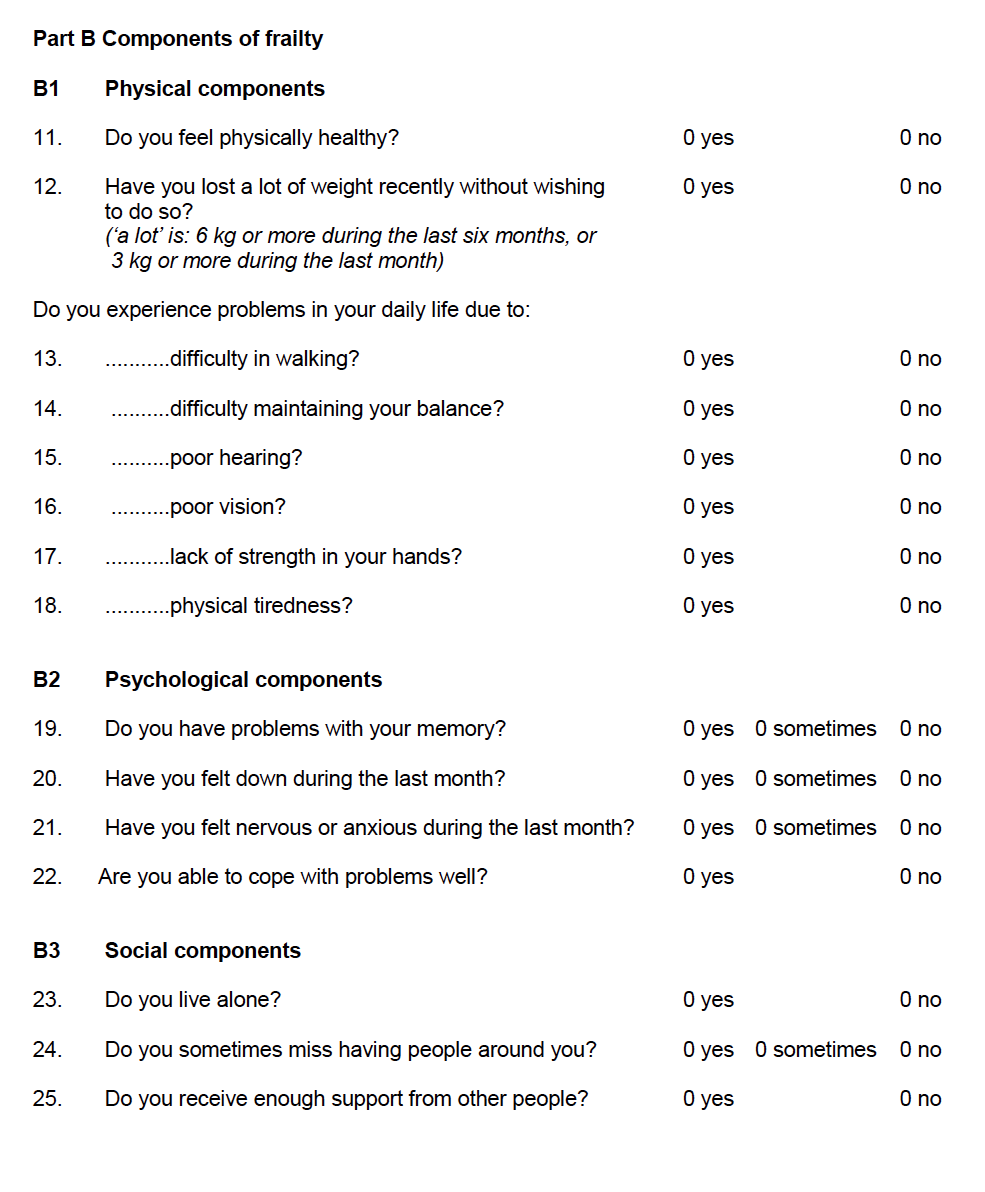

Supplement: Supplementary file 1 — Additional file 1. [file 12877_2020_1875_MOESM1_ESM.docx]
